# A Critical Review of LET-Based Intensity-Modulated Proton Therapy Plan Evaluation and Optimization for Head and Neck Cancer Management

**DOI:** 10.14338/IJPT-20-00049.1

**Published:** 2021-06-25

**Authors:** Wei Deng, Yunze Yang, Chenbin Liu, Martin Bues, Radhe Mohan, William W. Wong, Robert H. Foote, Samir H. Patel, Wei Liu

**Affiliations:** 1 Department of Radiation Oncology, Mayo Clinic, Phoenix, AZ, USA; 2 Department of Radiation Oncology, National Cancer Center/National Clinical Research Center for Cancer/Cancer Hospital & Shenzhen Hospital, Chinese Academy of Medical Sciences and Peking Union Medical College, Shenzhen, Guangdong, China; 3 Department of Radiation Physics, The University of Texas MD Anderson Cancer Center, Houston, TX, USA; 4 Department of Radiation Oncology, Mayo Clinic, Rochester, MN, USA

**Keywords:** linear-energy-transfer, intensity-modulated proton therapy, relative biological effectiveness

## Abstract

In this review article, we review the 3 important aspects of linear-energy-transfer (LET) in intensity-modulated proton therapy (IMPT) for head and neck (H&N) cancer management. Accurate LET calculation methods are essential for LET-guided plan evaluation and optimization, which can be calculated either by analytical methods or by Monte Carlo (MC) simulations. Recently, some new 3D analytical approaches to calculate LET accurately and efficiently have been proposed. On the other hand, several fast MC codes have also been developed to speed up the MC simulation by simplifying nonessential physics models and/or using the graphics processor unit (GPU)–acceleration approach. Some concepts related to LET are also briefly summarized including (1) dose-weighted versus fluence-weighted LET; (2) restricted versus unrestricted LET; and (3) microdosimetry versus macrodosimetry. LET-guided plan evaluation has been clinically done in some proton centers. Recently, more and more studies using patient outcomes as the biological endpoint have shown a positive correlation between high LET and adverse events sites, indicating the importance of LET-guided plan evaluation in proton clinics. Various LET-guided plan optimization methods have been proposed to generate proton plans to achieve biologically optimized IMPT plans. Different optimization frameworks were used, including 2-step optimization, 1-step optimization, and worst-case robust optimization. They either indirectly or directly optimize the LET distribution in patients while trying to maintain the same dose distribution and plan robustness. It is important to consider the impact of uncertainties in LET-guided optimization (ie, LET-guided robust optimization) in IMPT, since IMPT is sensitive to uncertainties including both the dose and LET distributions. We believe that the advancement of the LET-guided plan evaluation and optimization will help us exploit the unique biological characteristics of proton beams to improve the therapeutic ratio of IMPT to treat H&N and other cancers.

## Introduction

Particle (proton and heavy ion) radiation therapy is a rapidly evolving technology. Compared with conventional photon-based radiation therapy, particle therapy has a significantly lower entrance to peak dose ratio, since most of the particle energy is released near the end of its range (Bragg peak). This behavior offers improved conformal dose coverage of target volumes and reduces dose to organs at risk (OARs) in the low- to middle-dose range (eg, the low-dose bath effect) [1, 2], which potentially leads to less normal tissue damage and lowers the risk of secondary cancers. Meanwhile, from the radiobiology point of view, the high linear-energy-transfer (LET) behavior of particle therapy has less oxygenation dependency [3, 4] and can overcome the radioresistance of hypoxic tumor cells [5, 6].

Although the qualitative benefits of particle therapy are well known, there are still many challenges in its clinical application, such as the variable relative biological effectiveness (RBE) [7, 8]. RBE is defined as the ratio of doses to reach the same level of clinical endpoints by a radiation modality, such as proton therapy, as compared to Co-60 irradiation. Via RBE, we can take advantage of the extensive experience from photon treatment; otherwise extensive independent clinical data for particle therapy are required to generate dosimetric indices as a surrogate for potential clinical outcomes. Unfortunately, RBE of particle therapy has not been well quantified [7, 9–12] and is dependant on many factors, for example, LET, clinical endpoints, tissue type, fractionation scheme, patient-specific radiosensitivity, physical dose [7, 8], and the uncertainties in experimental measurements [13–18]. Some RBE models have been used in current clinical practice for both proton and heavy ion therapy. For proton therapy, a fixed RBE of 1.1 is applied clinically, which likely oversimplifies the RBE model and underestimates RBE around high LET locations (eg, distal end of Bragg peak) [19] in proton therapy. An underestimation of RBE will potentially increase the risk of toxicities in the nearby OARs.

From now on, in our discussion we will focus on intensity-modulated proton therapy (IMPT), the most contemporary generation of proton therapy available in current clinical practice. However, many concepts and research reviewed here are highly relevant to other heavy ion therapy modalities as well. Although there are many uncertainties, RBE of protons monotonically increases with LET when all other parameters are held constant [13, 20]. For protons, when LET is high (around 10 keV/μm) near the distal end of the proton beam and in the corresponding lateral penumbra regions, owing to multiple Coulomb scattering [7], RBE can be much higher than 1.1 (around 1.5). There are reports [21–23] of an increasing risk of brainstem, lung, and brain injury treated by proton therapy with increasing LET, suggesting that the proton RBE can exceed 1.1 in vivo. Hence, it is essential to develop an LET-based plan evaluation and optimization method for proton therapy. This issue is especially relevant in cancer treatment of disease sites such as head and neck (H&N), where there are many OARs such as brainstem and optic-nerve structures in the proximity [24–26].

In this report, we will review and summarize some classic and recent studies including LET calculation methods (analytical and Monte Carlo [MC] simulation; “LET Calculation” section), use of LET in H&N plan evaluation and patient outcome study (“Use of LET in H&N Plan Evaluation and Patient Outcome Study” section), and LET-guided plan optimization and implementation (“LET-Guided Plan Optimization and Implementation” section) for H&N cancer treatment by IMPT.

## LET Calculation

As discussed in the “Introduction” section, the LET value is a good surrogate of RBE [13, 20]. For any studies related to LET-guided plan evaluation and optimization in H&N irradiation, an accurate and sufficiently fast LET calculation method is required in clinical practice. We will briefly review 3 topics related to LET calculation in the following 3 subsections: (1) analytical methods versus MC simulations; (2) dose-weighted average (LET_D_) versus fluence-weighted average (LET_f_); and (3) macroscopic versus microscopic dosimetry.

### Analytical Methods versus MC Simulations

Monte Carlo simulations [11, 27–30] are generally more accurate than analytical approaches, but require much longer calculation time if a general-purpose MC code is used (eg, Geant4 [27], MCNPX [31], FLUKA [32, 33], and TOPAS [34]). Recently, several groups have developed in-house and/or open-source fast MC codes and implemented them into clinical practice, for example, gMC [35], gPMC [36], FRED [37], and MCsquare [38, 39]. These fast MC codes either simplify physics models dedicated to proton dose calculation or are accelerated with the help of graphics processor unit (GPU) to significantly reduce the simulation time with reasonable accuracy. Some commercial treatment planning systems (TPSs) with fast MC calculation capability have recently been released for routine dose calculation (eg, RayStation [40], Eclipse [41]) [42]. Meanwhile, some groups have also developed MC-based robust optimization [43, 44] and MC-based robustness evaluation [45]. However, the LET calculation based on fast MC has not been used in any commercial TPSs for clinical use.

Various techniques are used to score LET in MC simulations; for example, scoring LET using the ratio of energy deposition to step-length [10, 11, 46], using the prestep proton kinetic energy [28, 29], converting proton energy spectrum to LET spectrum [47–50], and so on. Granville and Sawakuchi [29] did a detailed comparison among different LET scoring methods and found LET_D_ varied more than LET_f_, using different scoring methods.

On the other hand, analytical LET calculation methods [15, 51–56] have been used in clinical practice owing to their high efficiency, acceptable computational accuracy, and other historical reasons. The widely used 1D LET models [13, 51, 52] only consider the LET variation along the beam direction and assume uniform LET values in the lateral direction. Since the LET value will significantly increase in the lateral distance from the beam axis owing to the higher proportion of low-energy halo particles, the 1D LET models underestimate LET in the penumbra region [55, 56]. Unfortunately the nearby OARs are usually located within the dose penumbra regions, therefore the LET values are likely underestimated within OARs, which potentially results in unexpected toxicities.

To overcome this drawback, Hirayama et al [55] and Deng et al [56] introduced 3D LET calculation models, which consider the variation of LET in the lateral directions. Hirayama et al [55] use a dual-Gaussian LET calculation kernel, which models contributions from the primary and the halo components of the proton beam separately. Deng et al [56] introduced a hybrid 3D analytical LET_D_ calculation approach based on the convolution superstition method commonly used in the pencil beam dose calculation. An accurate 3D analytical LET kernel by fitting the MC-simulated LET distribution was used.

### Dose-Weighted Average (LET_D_) versus Fluence-Weighted Average (LET_f_)

There are 2 approaches to calculate LET values. The first is called *dose-weighted LET* (LET_D_), which uses the dose of every proton beamlet as the weighting factor to average LET value within each voxel. The LET_D_ (*x*, *y*, *z*) at the voxel (*x*, *y*, *z*) can be calculated as follows:

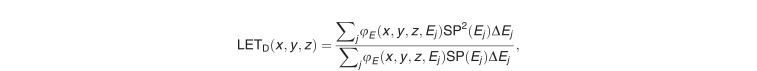
where *φ_E_*(*x*, *y*, *z, E_j_*) is the proton energy spectrum with an energy of *E_j_*, and SP(*E_j_*) is the unrestricted stopping power of protons with an energy of *E_j_*. The other is called *fluence-weighted LET* (LET_f_), which uses the fluence of every proton beamlet as the weighting factor to average LET values within each voxel (*x*, *y*, *z*) as follows:

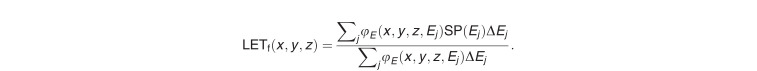
Although LET_D_ may have a better correlation with RBE, since LET_f_ tends to underestimate the biological effect, there are no rigorous comparisons to demonstrate which is superior.

Meanwhile, to be better correlated with biological effect, the kinetic energy of secondary δ-rays should be taken into consideration, since this energy, although transferred from the primary particles, is not deposited locally and will not affect the local RBE. Hence, the restricted LET_D_, which takes off the kinetic energy of secondary δ-rays, was discussed and compared with the unrestricted LET_D_ (ICRU 2016) in the literature [28, 57]. From the reported results, the restricted LET_D_ shows an apparent dependence with the cutoff energy. There is no clinical evidence so far to show that the restricted LET_D_ correlates with RBE better than unrestricted LET_D_.

### Macrodosimetry versus Microdosimetry

The concept of LET commonly discussed in the literature and used in clinical practice is in the scope of macrodosimetry, which is either the dose- or fluence-averaged LET corresponding to the voxel size (eg, millimeter scale). This approach is relatively easy to implement and is efficient to calculate. However, the concept of LET in the domain of macrodosimetry might compromise the correlation of LET to the biological effects in the microscopic domain and cannot reflect the potentially higher LET/RBE values from the microscopic point of view (eg, micrometer scale), since cells are much smaller than the millimeter scale. Hence, several groups have introduced the dose-mean lineal energy [58–62] (a counterpart concept of LET in the scope of microdosimetry).

Since it considers the microscopically imparted energy patterns instead of the macroscopically voxel size averaging, the dose-mean lineal energy will likely reflect the biological effects better than LET. Several groups have developed a microdosimetric–kinetic model [59, 60] and the subsequent modification models [61, 62] to describe the microdosimetric biological effect due to protons. Recently, models to calculate dose-mean lineal energy [57, 63, 64] have been developed and proposed to be used for the microscopic biophysical optimization [65, 66] in particle therapy. In the following section, we will give a more detailed review of different LET-related RBE models and their applications in plan evaluation and patient outcome study for IMPT to treat H&N cancer.

## Use of LET in H&N Plan Evaluation and Patient Outcome Study

### Relative Biological Effectiveness—as a Function of LET

Since proton LET increases at the distal end and RBE has a strong correlation with LET value, LET has been considered as one of the key factors for determining the possible variable RBE in proton therapy. A variety of LET-related RBE models have been proposed, including phenomenological models [8,13–16,46,67–73], which are mostly based on linear-quadratic models, and mechanistic models, such as the microdosimetric-kinetic model [60], the local effect model [74–76], and the repair-misrepair-fixation model [77]. We refer our readers to previous reviews for details of these models and their comparisons [7, 78, 79].

While these models were derived from multiple measurements of various biological endpoints, including in vitro ones such as clonogenic cell survival and DNA breaks, as well as in vivo ones such as those from small animals, we will exclusively focus our discussion on recent studies that use clinical data as the biological endpoints for dosimetric study of IMPT plan evaluation and for patient outcome study in patients with H&N cancer.

### Dosimetric Study Using LET for IMPT Plan Evaluation

Dosimetric evaluation of LET has been increasingly implemented in proton centers as a second check tool for IMPT. Typically, an analytically calculated or MC-simulated LET distribution of a treatment plan is generated for review. Physicians or physicists would evaluate the biological effects of the IMPT plan either by checking the overlapping volume of both high dose and high LET to avoid the overlap of dose and LET extremities in OARs, or by assessing the biological dose distribution derived from dose and LET distributions according to different RBE models. The Figure shows the LET distribution of a pediatric patient with skull base cancer treated by IMPT. High LET values above 6 keV/μm (shown as colorwash) were observed within brainstem in the original plan (*left* column) during the LET-guided plan evaluation for this patient. We therefore generated a new plan by using different beam angles (indicated as *red* arrows in the Figure) to minimize the high LET in brainstem. With similar dose distributions, the final plan (*right* column) displayed significant reduction of high LET distribution within brainstem. Currently this LET-guided plan evaluation has been routinely performed for every patient treated by IMPT at our institution.

**Figure.**
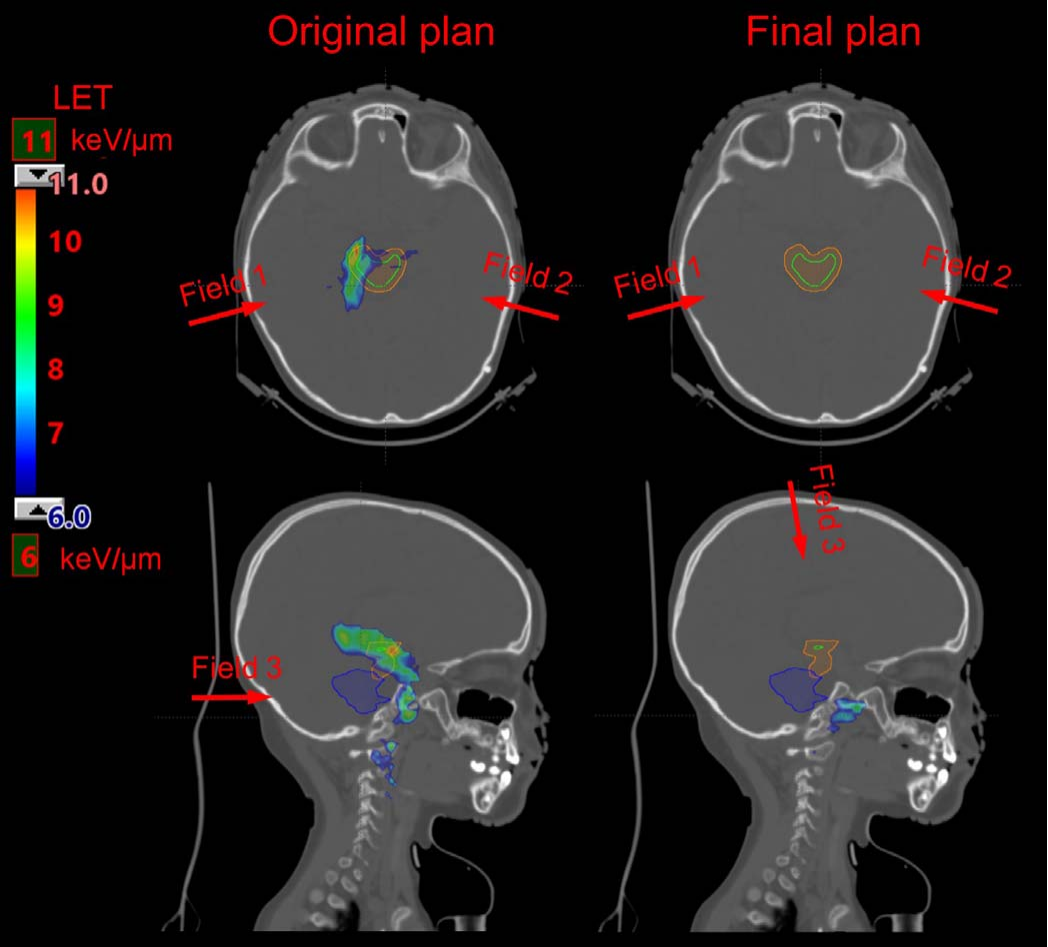
Linear-energy-transfer (LET) distribution in a pediatric patient with skull base cancer. LET distribution of original plan and of final plan (right column) after changing the angle of 1 field (left column, transversal [top row] and sagittal [bottom row] view). LET is displayed at a range of 6 keV/μm to 11 keV/μm. Significant high LET reduction was observed in brainstem (orange contour) and the core of brainstem region (green contour). Blue contour indicates contoured clinical target volume (CTV) region. Red arrows indicate beam angles.

Ideally, this plan evaluation procedure relies on (1) accurate and fast calculation of LET distributions (as discussed in “LET Calculation” section); (2) accurate biological dose calculation according to LET-related RBE models, and (3) reliable LET-related volume constraints (as dose volume constraints [DVCs] widely used in radiation therapy for decades). However, the last 2 criteria are currently unavailable in clinics, thus the second check is entirely based on estimation and experience from physicians and physicists.

Multiple studies have been carried out to evaluate the dosimetric effects of different RBE models in passive scattering proton therapy [80], and IMPT plans for numerous tumor sites including H&N [81–85], thorax [84, 86], liver [81], prostate [84, 87, 88], and craniospinal irradiations [89]. Tilly et al [68] first reported the influence on the dose volume histograms after RBE correction for a patient with hypopharynx cancer. Carabe et al [81] studied the impact of tissue-related parameter α/β and fractionations to biological dose, and revealed significant RBE uncertainties in critical structures for the central nervous system malignancies.

Comparison of different variable RBE models and evaluation of their influence have been studied in 2 clinical cases [82]. Giantsoudi et al [83] systematically studied LET and RBE for patients with medulloblastoma (111 patients). Another systematic study [84] was carried out for comparison of fixed versus variable RBE (including 80 patients with brain tumor and 128 with H&N cancer) with a variety of RBE models. Both studies indicated increased RBE doses at nearby OARs, compared to that using fixed RBE of 1.1. In addition, compared to planning target volume (PTV)–based optimization, robust optimization plans showed that dose distributions calculated with fixed-RBE of 1.1 were closer to the biological doses calculated with variant RBE models [85]. All these studies revealed dosimetric uncertainties of using different RBE models, as well as possible interpatient variations when applying RBE models. However, there is no consensus so far as to which model is better in RBE estimation.

### Patient Outcome Study Using LET in H&N IMPT

Despite numerous in vitro measurements and several in vivo studies that have been performed, it is unclear whether the derived RBE models are applicable to clinical patient outcomes as the biological endpoint. In fact, clinical investigations showed inconclusive results regarding the impact of LET in patient outcomes. Sethi et al [90] pioneered the study of the LET effect on tumor recurrence of medulloblastoma. No correlation between regions of disease recurrence and low LET was observed in 16 patients with tumor relapse. The complication of central nervous system injury over a similar patient cohort was also investigated [83]. Although elevated LETs were observed in 10 injured areas that were identified and contoured with the help of magnetic resonance imaging (MRI) after the treatment by IMPT, no significant correlation was observed between the injury sites and the high RBE value. Note that both studies compared the calculated RBE values at the recurrence/complication regions. The analysis might be affected by the uncertainties in the RBE model calculation and the statistical analysis, since the conclusions were derived simply from the mean value.

Other studies have evaluated the correlation of patient outcomes with the local LET distributions. In a study of 34 pediatric patients with ependymoma, the treatment responses of voxels at the injured sites (indicated by image changes in the follow-up MRI scans) were assessed and modeled with a linear combination of the dose and LET [23].The strong correlation between the response and the linear combination of the dose and LET strongly suggested a variable proton RBE in clinical practice. This finding was further supported by an analysis of asymptomatic lung fibrosis in chest-wall patients [22]. Results strongly suggested that the proton RBE exceeds 1.1, resulting in increase in lung fibrosis. A similar conclusion was drawn from recent studies in complications of radiation-induced cerebral vasculopathy in pediatric patients with craniopharyngioma [91] and multiple complications associated with intracranial tumors [92]. These findings were consistent with a variable RBE in vivo.

All the patient outcome studies summarized here exclusively rely on accurate contours of local injury/toxicity regions or functional regions at suborgan level that are typically obtained from the follow-up computed tomography and MRI images. Voxelwise tissue responses are correlated with the dose and LET values. In recent studies of glioma [93, 94], multivariable logistic regression models were established, with inputs not only from the dose and LET, but also from the interaction term such as the product of the dose and LET; and anatomy-related variables, such as the labeling of periventricular region, which indicates the proximities to the segmented ventricles as a risk factor. From this approach, a high correlation was observed in local complications of radionecrosis and late brain toxicities in relation to these factors. Moreover, a patient-level normal tissue complication probability (NTCP) model can be built with the voxel-level multivariate regression method by assuming that the relevant OARs are serial organs in the complication probability modelling [95]. This 2-level model achieved good local complication prediction and good NTCP models in the patient population for late radiation-induced contrast-enhancing brain lesions of a cohort of 110 patients with glioma. These recent works have provided direct clinical evidence that RBE increases significantly with LET. Thus various LET-guided plan optimization approaches have been proposed to mitigate the negative impact of high RBE/LET in OARs, as reviewed in the following section.

## LET-Guided Plan Optimization and Implementation

Various LET/RBE-guided plan optimization approaches have been proposed. Some pioneering works have been summarized here. Bassler et al [96] introduced the LET-painting technique in water phantom studies by using different irradiation modalities and beam configurations. The proton and carbon ion treatment planning system (TRiP) was used to yield treatment plans in conjunction with the in-house–developed software package “pytrip” [96]. The results demonstrated that similar dose distributions might have significantly different LET distributions. Grassberger et al [10] first investigated the LET distributions in patients by using MC simulations. The research version of KonRad generated 3-dimensional modulation IMPT and distal edge tracking IMPT [10]. Their results demonstrated that IMPT could have different LET distributions with clinically equivalent dose distributions, and the preselection of beam spots could be incorporated into the inverse-planning optimization to yield a superior LET distribution [10].

In the early stage of LET-guided optimization development, 2-step IMPT optimization methods were proposed to achieve a superior LET distribution [8, 97]. In the first step, dose distribution was optimized to meet current clinical requirements, and LET distribution was modified in the following step. Unkelbach et al [8] proposed a 2-step IMPT optimization scheme. The initial IMPT plans were optimized on the basis of physical dose, and then a prioritized optimization was used to modify the LET distribution and limit the degradation of physical dose distribution in the initial plan. Giantsoudi et al [97] also presented a dose-based multicriteria optimization method, which was integrated into the in-house treatment planning system (Astroid), to produce multiple IMPT treatment plans for navigating the Pareto-optimal space. In the second step, dose and LET distributions were calculated with the TOPAS (tool for particle simulation) MC method to facilitate the decision-making process [97]. Both methods were able to achieve superior LET distributions while maintaining equivalent dose distributions.

Instead of 2-step optimization, recent studies aimed to simultaneously optimize dose and LET distribution in the inverse planning for proton therapy [17, 73, 98–103]. Wan Chan Tseung et al [35, 73] developed a fast and accurate GPU-based MC method for LET-guided plan optimization in IMPT [73]. The LET distribution was integrated into a GPU-accelerated IMPT optimizer, which allowed the escalation of the target biological dose [73]. Cao et al [98] added 2 constraint terms for maximizing target LET and minimizing OAR LET in the cost function for optimization. Charnes and Cooper variable transformation was used to reformulate the original quasiconvex problem—which required sophisticated linear fractional programming—to a linear programming problem. The approach used by Cao and colleagues [98] did not use dose-volume/LET-volume constraints owing to the limitation of linear programming [104, 105]], thus the determination of priority factors for dose and LET constraint terms was required. To customize the optimization in a user-friendly way, Inaniwa et al [101] used the summation of least-square terms in the dose- and dose-weighted-LET–based cost function. The customized treatment planning system (IMPACT) allowed the dose-volume and LET-volume constraints independently [101]. In summary, the 1-step optimization allowed for dose and LET optimization simultaneously, which could search in a larger solution space than the 2-step optimization method, possibly leading to better-quality IMPT plans.

Proton therapy is very sensitive to range and setup uncertainties. Without considering these uncertainties, both the delivered dose and LET could be significantly degraded compared to the planned ones. Robust optimization is an efficient way to hedge against the negative impacts of the range and setup uncertainties [1, 2, 17, 102, 106–122]. An et al [17] extended the voxelwise worst-case robust optimization by adding constraint terms to restrict the biological effect of LET in OARs. The unconstrained convex programming problem was solved by the L-BFGS (limited-memory Broyden-Fletcher-Goldfarb-Shanno) algorithm in a parallel computation environment [17]. Similarly, Bai et al [99] added constraint terms of biological effects for both OARs and targets in voxelwise worst-case robust optimization to maximize the biological effect itself and the robustness of biological effects. The new model reduced the biological effects in OARs and the variations of biological effects in targets and normal tissues [99]. Recently, Liu et al [102] developed LET-guided robust optimization (LETRO), which simultaneously minimized the LET in OARs, maximized LET in targets, and considered the range and setup uncertainties. The proposed LET-volume constraints provided a user-friendly way for treatment planning, which is similar to DVCs widely used for decades in radiation therapy treatment planning. Compared with the conventional voxelwise worst-case robust optimization, LETRO slightly escalated the target LET and reduced the OAR LET by slightly sacrificing plan quality and plan robustness for H&N cancer [102]. It is worth noting that only a few studies directly used LET objectives in the cost function [97, 98, 101, 102]. In other studies, LET-related biological terms were used as an indirect indicator of LET in the optimization, including LET-weighted dose [8], biological surrogate [17], biological dose [73], biological component [99], and proton track ends [103].

Since the LET peak appears beyond the Bragg peak of proton beams, the optimization of proton beam angles and proton spot locations could render a superior LET distribution [97, 100, 103]. Traneus et al [103] used the location of the proton track-ends objectives in the IMPT treatment plan optimization. The method did not introduce additional complexity by using the LET calculation, LET-weighted dose, or LET-RBE interrelationship, but allowed for LET increase in target and LET reduction in OARs without sacrificing physical dose distribution. Bai et al [100] proposed a distal-edge avoidance-guided optimization method. Proton spots were categorized into 4 groups on the basis of topologic relationship among the peak position of LET-weighted dose, target location, and OAR location. The simplified calculation of peak position of LET-weighted dose used a certain distance away from the proton spots along the beam direction [100]. Then different groups of spots would use different penalty weights in the optimization [100]. Both studies optimized the proton spot locations instead of the LET-related objectives in the IMPT inverse treatment planning and achieved a superior LET distribution in both target and OARs [100, 103]. Traneus et al [103]. However, LET distribution was not explicitly optimized [100]. The tedious trial-and-errors iterations to adjust the penalty weights for dose and proton spot location terms were required.

## Discussion and Future Work

During the last 10 years, significant progress has been made in LET-related research in proton and heavier-particle therapy with regard to LET calculation, LET-guided plan evaluation, and LET-guided optimization. Many in vitro and in vivo studies have demonstrated the importance of the LET and variation of RBE in proton therapy, especially for H&N cancer. Recently, the TG-256 report [19] suggested assessing the potential clinical consequences, based on LET and recommended LET-guided optimization in IMPT.

In this review, we have focused on the advantages and disadvantages of different LET calculation methods from the points of view of accuracy, efficiency, and the relevance to biological effects in proton therapy. Highly accurate and efficient LET calculation methods based on either analytical methods or MC simulations are now available for clinical use, with the fast adoption of these methods in the commercial TPSs in the near future.

Currently, standard practice in most proton centers only checks for overlap of high dose and high LET in nearby OARs during the LET-guided IMPT plan evaluation with the goal of minimizing the overlap region. This simple method works relatively well although the thresholds of high dose and high LET are not based on clinical outcome. The LET-guided plan evaluation and patient outcome studies are done only in some academic centers. However, we expect that this will become the standard practice for IMPT to treat H&N cancer in the next 5 years. As more patient outcome data using IMPT become available and more LET-related patient outcome studies are performed, we believe that some LET-related volume constraints just as DVCs derived from QUANTEC [123] for plan evaluation purely based on the dose distribution will become available. The LET-related volume constraints can be used for plan evaluation and to predict the potential clinical consequences of IMPT plans in all disease sites with OARs abutting targets.

Today, no clinical studies with LET-guided optimization have been reported. It is important to consider the impact of uncertainties in LET-guided optimization (ie, LET-guided robust optimization) in IMPT, since IMPT is sensitive to uncertainties including both the dose and LET distributions. While some commercial TPSs such as the research version of RayStation (RayResearch Laboratories, Stockholm, Sweden) and some in-house–developed TPSs have LET-guided robust optimization implemented, more experience is needed before LET-guided robust optimization in IMPT can be adopted in clinical practice routinely. The relevant experience can also be used to treat some other disease sites as in LET-guided plan evaluation. More importantly, we can incorporate the LET-related volume constraints into the LET-guided robust optimization to achieve the patient-specific precision proton therapy for H&N cancer when the LET-related volume constraints are available. More research in this direction is urgently needed.

Accounting for increased biologic effectiveness of proton therapy in the treatment of H&N cancer is an unsolved problem. There is near universal agreement that the current approach of applying a fixed RBE of 1.1 is insufficient to prevent unwanted side effects of proton therapy and to model patient outcomes accurately. Furthermore, it is unclear whether any RBE model, which relies solely on physical dose, LET, and fractionation scheme, with the possible addition of some cell/tissue parameters, can accurately reflect the clinical reality of H&N cancer treatment with protons. What may be needed instead is a phenomenologic disease site– and organ-specific patient outcomes–oriented model that forgoes the computation of an equivalent photon dose along the lines of RBE, and based on physical dose, LET, fractionation scheme, and other factors, directly predicts probabilities for tumor control and healthy-tissue complication. Owing to the complexity of the problem at hand, such a model would require an artificial intelligence–based algorithm, training sets of several hundred if not thousands of cases per organ per disease site, and possibly stratification by known risk factors such as concurrent chemotherapy and other unknown risk factors. These unknown risk factors could be systematically explored in this approach.

We believe that the advancement of LET-guided plan evaluation and optimization will help us exploit the unique biological characteristics of proton beams to further improve the therapeutic ratio of IMPT to treat H&N and other cancers.
